# THE IMPACT OF IMPLEMENTING THE PREFERENCES FOR EVERYDAY LIVING INVENTORY (PELI) ON SURVEY DEFICIENCY OUTCOMES

**DOI:** 10.1093/geroni/igac059.919

**Published:** 2022-12-20

**Authors:** Katherine Abbott, John Bowblis, Jane Straker, Kimberly Van Haitsma

**Affiliations:** Miami University, Oxford, Ohio, United States; Miami University, Oxford, Ohio, United States; Scripps Gerontology Center at Miami University, Oxford, Ohio, United States; Pennsylvania State University, University Park, Pennsylvania, United States

## Abstract

The Ohio Department of Medicaid added the PELI as one of five quality incentive points used to determine Medicaid per diem reimbursement rates with the goal of improving person-centered care among Ohio’s nursing homes (NHs). The purpose of this study was to explore if the degree of PELI implementation had an impact on quality as defined by deficiencies and star rating. Multinomial logistic regression of 1,300 NH-year observations was used to understand the relationships between survey star ratings/deficiency scores on PELI implementation controlling for organizational characteristics of the NH. We find complete PELI implementation statistically significantly increased the probability of being a 4- or 5-star survey-rated facility. Complete PELI implementation is associated with small improvements each year that add up. Organizational change takes commitment and persistent effort over time to see changes in quality as reflected by star ratings and deficiencies. Recommendations for practice and policy will be discussed.

